# IRIXS Spectrograph: an ultra high-resolution spectrometer for tender RIXS

**DOI:** 10.1107/S1600577521003805

**Published:** 2021-05-27

**Authors:** Joel Bertinshaw, Simon Mayer, Frank-Uwe Dill, Hakuto Suzuki, Olaf Leupold, Atefeh Jafari, Ilya Sergueev, Manfred Spiwek, Ayman Said, Elina Kasman, Xianrong Huang, Bernhard Keimer, Hlynur Gretarsson

**Affiliations:** a Max-Planck-Institut für Festkörperforschung, Heisenbergstrasse 1, D-70569 Stuttgart, Germany; b Deutsches Elektronen-Synchrotron DESY, Notkestrasse 85, D-22607 Hamburg, Germany; c Advanced Photon Source, Argonne National Laboratory, Lemont, IL 60439, USA

**Keywords:** IRIXS beamline, PETRA III, DESY, RIXS, spectrograph, beamlines

## Abstract

The IRIXS Spectrograph represents a new design of an ultra-high-resolution resonant inelastic X-ray scattering (RIXS) spectrometer that operates at the Ru *L*_3_-edge (2840 eV). First proposed in the field of hard X-rays by Shvyd’ko [(2015), *Phys. Rev. A*, **91**, 053817], the X-ray spectrograph uses a combination of laterally graded multilayer mirrors and collimating/dispersing Ge(111) crystals optics in a novel spectral imaging approach to overcome the energy resolution limitation of a traditional Rowland-type spectrometer [Gretarsson *et al.* (2020), *J. Synchrotron Rad.* **27**, 538–544]. In combination with a dispersionless nested four-bounce high-resolution monochromator design that utilizes Si(111) and Al_2_O_3_(110) crystals, an overall energy resolution better than 35 meV full width at half-maximum has been achieved at the Ru *L*_3_-edge, in excellent agreement with ray-tracing simulations.

## Abstract 2

A resonant inelastic X-ray scattering (RIXS) spectrograph based upon Montel optics capable of energy resolutions better than 35 meV at the Ru *L*_3_-edge (2840 eV) has been constructed at beamline P01 of the PETRA III synchrotron.

## 1. Introduction   

Resonant inelastic X-ray scattering (RIXS) is a powerful technique capable of studying the momentum, energy and polarization dependency of intra- and inter-atomic charge, spin, orbital and lattice excitations of matter (Ament *et al.*, 2011 ▸). Studying magnons (Braicovich *et al.*, 2009 ▸) in particular requires instrumentation capable of accessing an energy transfer of less than 100 meV, which puts a great emphasis on resolving power. In this respect notable improvements in beamlines include the use of a spherical variable-line-spacing grating for the soft X-ray regime (*e.g.* Cu *L*_3_-edge, ∼940 eV) and a flat crystal analyzer for hard X-rays (Ir *L*_3_-edge, ∼11 keV), with the latter achieving a record Δ*E* < 10 meV resolution (Kim *et al.*, 2018 ▸).

The importance of ultra-high-resolution RIXS in the field of condensed matter physics cannot be overstated. While the current generation soft X-ray RIXS instruments (Δ*E* < 50 meV) (Brookes *et al.*, 2018 ▸) are providing new insight into spin (Peng *et al.*, 2017 ▸) and charge (Hepting *et al.*, 2018 ▸) excitations in superconducting cuprates, the hard X-ray RIXS instruments (Δ*E* < 30 meV) (Moretti Sala *et al.*, 2018 ▸) have opened a unique window into unconventional magnetism in iridates (*e.g.* Hwan Chun *et al.*, 2015 ▸). Despite these very exciting results, ultra-high-resolution RIXS in the intermediate (tender) energy regime (2.5–3.5 keV) has remained out of reach, making it difficult to study such complex dynamics in 4*d*-systems (*e.g.* Ru-based compounds). The problem is inherently multifaceted — in this energy range not only is the resolving power limited but the reflectivity of optical elements suffers. A realistic grating (1200 lines mm^−1^) based on today’s technology gives a resolving power of <20.000 (>140 meV) at the Ru *L*_3_-edge (2.840 keV), and a reflectivity of only a few percent (Viefhaus *et al.*, 2013 ▸), although multilayer gratings show promise to eventually overcome this limitation (Senf *et al.*, 2016 ▸). For crystal-based optics, on the other hand, the relatively long X-ray wavelength severely limits the available Bragg reflections. For instance, silicon, the most common material for monochromators due to its excellent crystalline quality and low thermal expansion coefficient, only offers the (111) Bragg reflection at the Ru *L*_3_-edge, which has an intrinsic bandwidth of ∼370 meV. Moreover, a far lower reflection efficiency (∼70% at 3 keV versus ∼100% at 10 keV) strongly impacts the flux–resolution trade-off as multiple optical elements are introduced. Lower symmetry crystals, like quartz and sapphire, offer an order of magnitude more unique Bragg reflections. They tend to be unstable under heat load, which can in some cases lead to degradation when exposed to a broadband beam (Gog *et al.*, 2018 ▸), limiting their use as monochromating elements. On the other side, they have been used successfully as hard X-ray analyzers in back-scattering geometry (Yavaş *et al.*, 2007 ▸, 2017 ▸; Ketenoglu *et al.*, 2015 ▸; Said *et al.*, 2018 ▸; Kim *et al.*, 2018 ▸).

The intermediate X-ray energy RIXS instrument IRIXS (Gretarsson *et al.*, 2020 ▸), located at beamline P01 of the PETRA III synchrotron in Hamburg, Germany, has successfully implemented a hard X-ray optic type layout for high-resolution Ru *L*_3_-edge RIXS (Suzuki *et al.*, 2019 ▸, 2020 ▸; Gretarsson *et al.*, 2019 ▸; Bertinshaw *et al.*, 2021 ▸). It uses Bragg reflections from asymmetrically cut Si(111) single crystals in a dispersive four-bounce monochromator, and a SiO_2_( 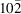 ) spherically bent and diced analyzer, giving an overall resolution of ∼75 meV full width at half-maximum (FWHM). This value approaches the theoretical limit set by the Darwin width of the quartz analyzer, necessitating the need for an entirely new approach to reach better energy resolutions. Recent ultra-high-resolution RIXS at the Ir *L*_3_-edge illustrated a promising approach through the addition of *collimating* optics (Kim *et al.*, 2018 ▸), but this does not address our intrinsic energy bandwidth issue. Here we introduce an alternate collimating approach that implements the *spectrograph* concept, where the resolving power relies on the cumulative angular dispersion rate of the spectrometer, instead of the Darwin width of the analyzer crystal (Shvyd’ko, 2015 ▸). Moreover, unlike a conventional analyzer, which by design selects only a small portion of a polychromatic beam, the spectrograph can capture the full incident beam in a single measurement, dramatically enhancing the number of photons that can be detected.

The key component of a spectrograph is the dispersive element. In the tender or hard X-ray regime, this element can consist of one or more asymmetrically cut crystals, which introduce an angular dispersion to the Bragg reflected X-rays. As a result, different energies have different take-off angles, similar to an optical prism (Shvyd’ko *et al.*, 2006 ▸). This energy dispersion can then be combined with a focusing mirror (or a lens) to map different energies spatially (Shvyd’ko, 2015 ▸; Kohn *et al.*, 2009 ▸). Such a scheme has been demonstrated and implemented as a high-resolution monochromator (HRM) for nuclear inelastic scattering (Shvyd’ko *et al.*, 2013 ▸; Chumakov *et al.*, 2019 ▸), but to our knowledge an X-ray spectrometer utilizing the spectrograph concept has not previously been reported.

In this article we present our Ru *L*_3_-edge IRIXS Spectrograph spectrometer and its method of operation, illustrated through ray tracing, and provide experimental data of its operating performance. Our design uses a laterally graded parabolic Montel mirror in a combination with collimating and dispersing multi-crystal optics. This setup gives the photons entering the spectrometer a large energy dispersion rate of 11 µrad meV^−1^, which is then converted into real space coordinates onto a position-sensitive detector (PSD) using a second Montel mirror. The IRIXS Spectrograph captures a 120 meV spectral window in a single shot, resulting in an excellent throughput. In combination with a novel narrow bandwidth (30 meV FWHM) dispersionless nested four-bounce high-resolution monochromator, we demonstrate an overall energy resolution better than 35 meV FWHM.

## 2. Overview of the instrument   

**Figure 1:**
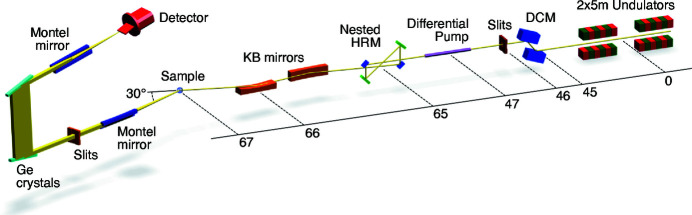
Layout of the complete IRIXS Spectrograph instrument at P01 showing the position of various beamline components (in meters) with respect to the center of the undulators. The beam propagates from right to left, going through multiple elements before hitting the sample, with the subsequent spectral response collected by the Spectrograph (see text for details). For clarity the items are not drawn to scale.

Figure 1 ▸ shows a schematic layout of beamline P01 that focuses on components relevant to the IRIXS Spectrograph. The beamline, which is entirely windowless and operates at a vacuum level of <10^−6^ mbar, has an estimated flux of 1 × 10^14^ photons s^−1^ after the double-crystal monochromator (DCM) around 3 keV and with a bandwidth of 600 meV. More general information on beamline P01 as well as the components upstream of the differential pump can be found in Gretarsson *et al.* (2020 ▸). The items downstream of the differential pump are new to the spectrograph project. A four-bounce nested high-resolution monochromator (4B-HRM) replaces the previous in-line design, providing a factor of two improvement in bandwidth (30 meV) at 2.840 keV. A new Kirkpatrick–Baez (KB) mirror system then focuses the photons onto the sample with a spot size of 17 µm × 13 µm (H × V). This large system has a 600 mm-long horizontal focusing mirror and a 300 mm-long vertical focusing mirror, operating with 4 mrad and focal distances of 1.7 m and 1.1 m, respectively, in order to capture the entire beam. The momentum and energy-dependent scattered signal is collected by the spectrograph spectrometer, which encompasses two sets of Montel mirrors, two asymmetrically cut Ge(111) crystals and a PSD mounted at an almost grazing angle. A slit system was installed after the first Montel mirror for diagnostics and to suppress background noise. In the following sections we will describe in detail the design and performance of the nested HRM and the spectrograph. This new setup has been optimized for the Ru *L*_3_-edge RIXS and a possible extension to other edges is limited by the relatively narrow energy bandwidth of the Montel mirrors (2–5%). Characterization of the spectrometer was conducted using the the elastic scattered signal from a droplet of GE-varnish collect at a 2θ ≃ 30°. The instrument was designed and optimized with the help of the ray-tracing package *XRT* (Klementiev & Chernikov, 2014 ▸). All optical components were modeled from source to detector, and virtual screens were positioned at each component to track the energy spectrum, physical size and divergence of the X-ray beam as it propagates through the instrument.

## 3. High-resolution monochromator   

To fully take advantage of the spectrograph’s potential a new HRM design was implemented. Since the collimation of the RIXS signal is proportional to the size of the focus on the sample (see discussion in next section[Sec sec4]), the HRM should not create a virtual source (*i.e.* increasing the angular divergence of the beam and therefore limiting the focusing ability) (Huang *et al.*, 2012 ▸). This latter point is a known problem in an in-line 4B-HRM design (Toellner, 2000 ▸; Yabashi *et al.*, 2001 ▸); an about seven-fold increase in the vertical divergence was observed by Gretarsson *et al.* (2020 ▸), increasing the vertical focal size to ∼150 µm. To avoid this issue we designed a nested 4B-HRM (Ishikawa *et al.*, 1992 ▸; Toellner *et al.*, 1993 ▸; Mooney *et al.*, 1994 ▸) where the two channel-cuts compensate for any added dispersion (Huang *et al.*, 2012 ▸).

**Figure 2:**
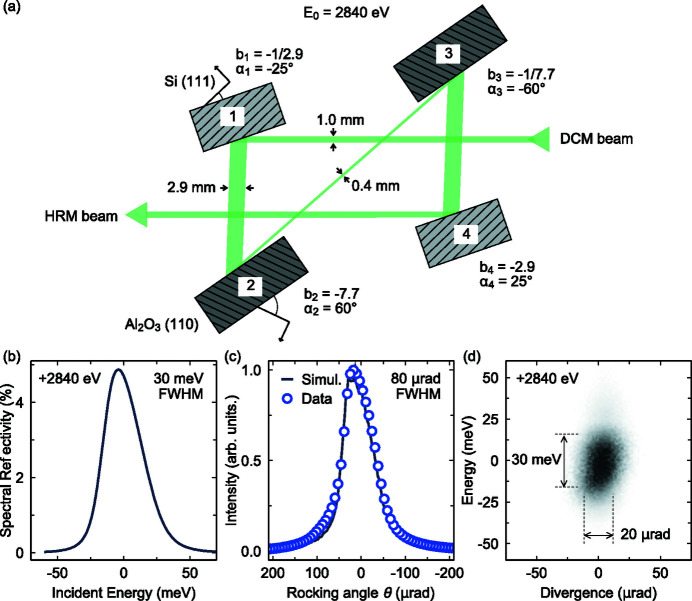
(*a*) Schematic diagram of the nested 4B-HRM. It consists of two artificial channel-cuts: Si(111) with asymmetry angles α_1_ = −25° and α_4_ = −α_1_, and Al_2_O_3_(110) (α_2_ = 60°, α_3_ = −α_2_). The first (silicon) reflection collimates the beam while the second (sapphire) selects the energy. The remaining crystals return the beam back to its original size and direction. (*b*) Simulated spectral reflectivity curve of the HRM showing an efficiency of 4.8%. (*c*) Comparison between the experimental rocking curve of crystal number 4 and simulations. (*d*) Calculated phase space of the photons exiting the HRM showing a well collimated beam with a 30 meV energy bandwidth.

In Fig. 2 ▸(*a*) we show a schematic diagram of our dispersionless nested 4B-HRM. It utilizes two artificial channel-cuts of Si(111) and Al_2_O_3_(110) crystals, facing each other to form a (+ + − −) configuration. The first crystal is Si(111) (Bragg angle θ_B1_ = 44.1°), with a surface cut away asymmetrically from the (111) planes at an angle α_1_ = −25°. The second crystal is Al_2_O_3_(110) with α_2_ = 60° (θ_B2_ = 66.6°). The asymmetry angles were carefully selected to maximize the energy resolution while still maintaining reasonable photon flux. The third and fourth crystals are inverted with respect to the first two, *i.e.* α_3_ = −α_2_ and α_4_ = −α_1_. The corresponding asymmetry parameters are 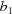 = 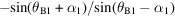 = −1/2.9, *b*_2_ = −7.7, *b*_3_ = −1/7.7 and *b*_4_ = −2.9. Following Δ*E* = Δ*E*_0_ × (1/|*b*|)^1/2^ (where *E*_0_ is the normal bandwidth at 2840 eV for a symmetric reflection) (Shvyd’ko, 2004 ▸), we estimate the bandwidth of the first silicon reflection to be Δ*E*_1_ = 650 meV and the first sapphire reflection to be Δ*E*_2_ = 50 meV. Similarly, the Darwin width of a Bragg reflection is also renormalized using (Δθ = Δθ_0_ × (1/|*b*|)^1/2^), while the reflected beam is collimated by a factor of |*b*| (Shvyd’ko, 2004 ▸). The bulk of the monochromatizating is therefore carried out by crystal 2, but the collimation of crystal 1 is vital to the overall performance. In addition, crystal 1 enlarges the beam, which spreads the radiation over a large area and minimizes any instabilities of the following Al_2_O_3_(110) crystals. Finally, since *b*_3,4_ are reciprocals of *b*_1,2_, the beam size remains the same upon exiting the HRM, albeit with a vertical offset of ∼10 mm.

We have calculated the performance of the nested-HRM more precisely using ray-tracing with *XRT*. Our model implements a geometrical source with a Gaussian profile centered at 2840 eV and a realistic vertical beam divergence (∼20 µrad, FWHM), which propagates through the DCM before reaching the HRM. In Fig. 2 ▸(*b*) we show the simulated spectral reflectivity, giving a FWHM 30 meV and a maximum reflectivity of 4.8%. We further test the performance of the HRM by rocking the final Si(111) crystal and comparing the experimental curve with simulations in Fig. 2 ▸(*c*). Data were collected using a diode detector placed directly after the HRM. The ray-tracing simulation shows that the Darwin width of crystal 4 is reduced from the symmetric value of ∼130 µrad to Δθ_4_ ≃ 80, in excellent agreement with our experimental result, indicating that the incident beam on crystal 4 is indeed highly monochromated and collimated. In Fig. 2 ▸(*d*) we plot the simulated energy and angular profiles of the beam after the HRM, which shows that the beam has an energy bandwidth of around 30 meV while maintaining the incident divergence of ∼20 µrad. This greatly enhanced performance over the previous in-line design (Gretarsson *et al.*, 2020 ▸) comes at the cost of a factor of three in efficiency, going from a value of 13% to 4.8%. This new design, as stated above, is however required in order to maintain the resolving power of the spectrograph. Taking into account the smaller bandwidth, the reduction in total photon flux when going from the in-line to the nested HRM is therefore a factor of six.

## 4. Spectrograph design   

**Figure 3:**
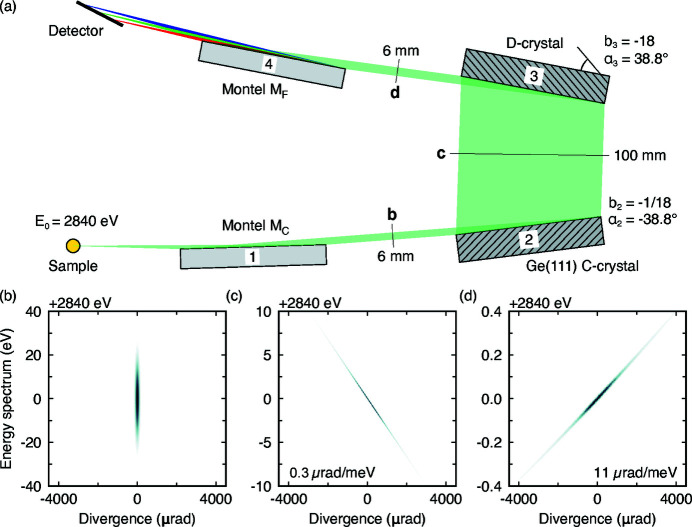
(*a*) Schematic diagram of the spectrograph. For simplicity the horizontal components of the Montel mirrors are omitted. Photons scattered from a sample are collected and collimated by a Montel mirror (M_C_) before being further collimated (C) and dispersed (D) by two Ge(111) crystals with asymmetry angles α_2_ = −38.8° (*b*_2_ = −1/18) and α_3_ = −α_2_ (*b*_3_ = −18). The acquired energy dispersion of the beam is then mapped onto a position-sensitive detector using a focusing Montel mirror (M_F_). This effect is presented as red, green and blue lines hitting the detector at three different positions. Ray-tracing simulations follow the phase space of photons at positions marked with black lines: (*b*) non-dispersive photons exiting M_C_, (*c*) after the C-crystal, which introduces a dispersion rate 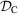 = 0.3 µrad meV^−1^, and (*d*) after the D-crystal, where the dispersion rate increases to 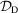 = 11 µrad meV^−1^.

With the upstream elements described, we now turn to the design of the IRIXS Spectrograph. The operating principle is illustrated in Fig. 3 ▸(*a*). The performance was optimized through ray-tracing simulations. Virtual screens were placed at the marked positions to give the results plotted in Figs. 3 ▸(*b*)–3(*d*). The first element is a collimating Montel mirror (M_C_, with a working distance of 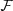 = 200 mm) that collects a large solid angle of the scattered signal from the sample and creates approximately a 100 µrad beam with a height of 5.5 mm [Fig. 3 ▸(*b*)]. The second element is a collimator (C), an asymmetrically cut Ge(111) crystal (α_2_ = −38.8°, *b*_2_ = −1/18, θ_B_ = 41.8°) with a large angular acceptance of ∼1300 µrad (Δθ_0_ = 300 µrad). It captures the entire post-Montel beam and collimates it further by a factor of |*b*_2_|, resulting in a beam with a divergence of ∼6 µrad and small dispersion rate of 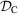 = 0.3 µrad meV^−1^, extending across Δ*E*_C_ = 4 eV [Fig. 3 ▸(*c*)]. At the same time the beam profile expands by a factor of 1/|*b*_2_| to ∼100 mm. The third element is a dispersive object (D), a Ge(111) crystal with the opposite configuration (α_3_ = 38.8°, *b*_3_ = −18). It accepts only 70 µrad and produces a highly dispersive beam 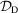 = 11 µrad meV^−1^ across Δ*E*_CD_ = 120 meV [Fig. 3 ▸(*d*)]. The beam size is also reduced back to 5.5 mm. We note that our reason behind using Ge(111), instead of Si(111), was that Ge(111) has more then twice the Darwin width of Si(111) and therefore our D element can accept a larger energy bandwidth.

Following the nomenclature of Shvyd’ko *et al.* (2013 ▸) the two germanium crystals form a CD-type (collimation-dispersion) multi-crystal optical element. In order to obtain a measurable spectrum, this element needs to be combined with either an angular analyzer (CD + W) to select a small portion of the inelastic signal, or a focusing mirror (CD + M_F_) to capture the entire spectrum (*i.e.* the spectrograph concept). The fourth element is therefore a second Montel mirror M_F_ with a configuration inverse to M_C_, in order to take the highly dispersive beam in Fig. 3 ▸(*d*) and focus ( 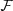 = 200 mm) it onto a spot in real space with a vertical size of 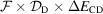 ≃ 260 µm. As discussed in the previous section, this results in an energy gradient of ∼2 µm meV^−1^, which can then be captured using a PSD. This poses a unique challenge, however, as the detector must be placed within the focal plane of the energy spectrum, which lies approximately 2° away from the outgoing focused beam [see discussion in Sanchez del Rio & Shvyd’ko (2019 ▸)]. A custom PSD was manufactured by Greateyes GmbH for this task. The ALEX detector implements a full-frame charge-coupled device (CCD) silicon detector chip with 2048 × 2064 pixels with a 15 µm pitch and no ceramic frame in the direction of the beam, giving a clear path for the photons to hit the CCD. We note that the 2° lies above the Si critical angle for total reflection for X-rays at 2840 eV, and that the penetration depth at this energy is large enough to pass through the detector chip SiO_2_ surface oxide layer. Based on this geometry, the 260 µm spot size spreads over 7.5 mm (500 pixels), giving a calculated dispersion rate of ∼60 µm meV^−1^ or ∼17 meV mm^−1^. In the following subsections we will detail the impact of manufacturing limits of the Montel optics and germanium crystals upon the performance of the IRIXS Spectrograph.

### 4.1. Montel optics   

A Montel mirror consists of two separate parabolic multilayer mirrors, joined orthogonally in an L-shape configuration along their edge, as depicted in Fig. 1 ▸. A well defined lateral grading in the period of the multilayers ensures that incoming rays with varying incident angle along the length of the mirror are Bragg reflected with equal efficiency. As a result, the Montel mirror collects and collimates a relatively large solid angle of scattered X-rays from a point source (or alternately focuses a well collimated beam if used in the reverse geometry) in both vertical and horizontal directions. The M_C_ and M_F_ mirrors were manufactured by AXO GmbH and Incoatec GmbH, respectively, and operate around the Ru *L*_3_-edge at 2840 eV. They nominally accept more than 30 mrad × 30 mrad of scattered radiation and collimate the beam to around 100 µrad × 100 µrad. The full set of manufacturer specifications are listed in Table 1 ▸.

**Figure 4:**
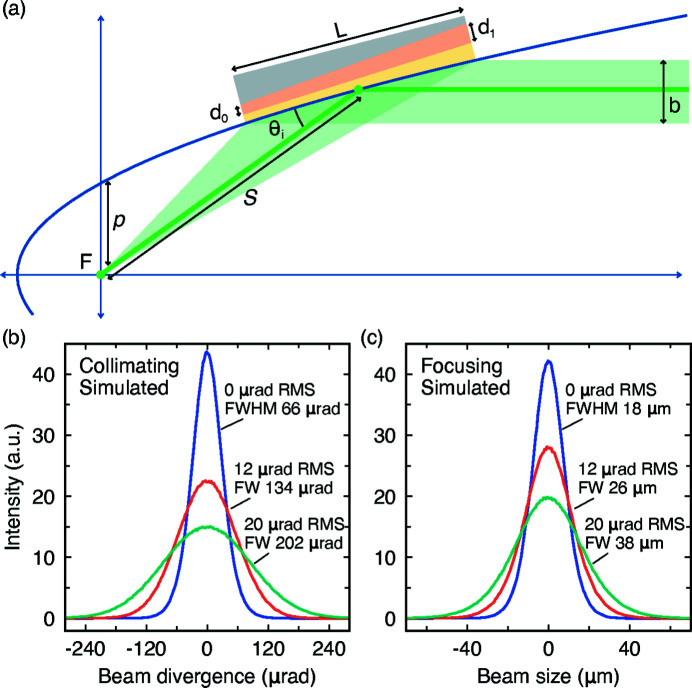
(*a*) Montel mirror schematic, illustrating the parameters that define the focusing and collimation performance. Only a single axis is shown for clarity; in reality a Montel mirror consists of two identical mirrors in an L-shape configuration to cover vertical and horizontal directions. (*b*, *c*) Realistic ray-tracing simulations of the Montel mirror element illustrate the impact of slope error (as root-mean-squared variance) on the collimation and focusing performance.

To quantify the expected performance of these mirrors we implemented a Montel optical element in *XRT* that takes into account the effect of slope error (the angular deviation of the surface curvature away from a perfect parabolic shape). The element, and therefore the entire ray-tracing model, was simplified to only operate in the beam diverging direction (*i.e.* the vertical scattering plane). We started by considering a parabolic mirror with a multilayer coating defined with the parameters in Table 1 ▸, and a *d*-spacing gradient corresponding to the incident Bragg angle along the surface of the mirror 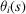 = 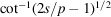 , where *p* is the vertex radius and *s* is the local coordinate along the paraboloid axis with origin at the vertex (F) [see Fig. 4 ▸(*a*)]. Slope error was implemented as root-mean-squared (RMS) variance by introducing a randomness to the local normal of the montel mirror element surface, which affects the outgoing vector of each reflected ray. *Twice* the magnitude of the quoted slope error was applied to take into account the effect of surface distortion upon the incoming and outgoing path of the rays (outgoing X-rays have an angle of 2θ). The effect of RMS slope error upon the collimation and focusing performance of the Montel mirror model is shown in Figs. 4 ▸(*c*) and 4(*d*). A 13 µm vertical sized source with a Gaussian distribution was used for the collimation while a 5.5 mm vertical source and 100 µrad collimated beam was used for the focusing part. In the case of a perfect parabola the collimation is limited by the source size, giving us around 66 µrad FWHM, which is in agreement with a simple back-of-the-envelope calculation, 0.013 mm/200 mm = 65 µrad. For focusing, the performance is, however, limited by the collimation, resulting in a spot size of ∼18 µm. Upon introducing the upper limit of the measured slope error, 12 µrad RMS (28 µrad FWHM), the collimation and focusing are broadened to 134 µrad (FWHM) and 26 µm, respectively. Moving from a slope error of 12 to 20 µrad (RMS) further degrades its performance, almost proportionally. Our simulations also show that the source size and the slope error contribute equally to the operation of the mirror, a favorable scenario since there is no one dominant limiting factor.

### 4.2. Germanium crystals   

The two germanium crystals were cut from a single boule and have identical dimensions; 150 mm long and a cross section of 10 mm × 15 mm (H × W). The relatively large size of the crystals stems from our requirement for aggressive collimation. For the IRIXS Spectrograph the combination of a large 5.5 mm post-M_C_ beam and the large 1/|*b*_2_| parameter results in an X-ray footprint that reaches almost 100 mm. This unfortunately also dramatically increases the probability of lattice gradient, which can strongly impact the overall energy resolution. Maintaining a uniform *d*-spacing across such a large area is challenging as factors such as residual mounting strain, thermal gradient, and defects become more evident at these length scales. During commissioning we indeed identified a large footprint issue with the crystals. While high-quality silicon is available with 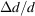 ≃ 1 × 10^−8^, even over an area of 60 mm × 60 mm (Fujimoto *et al.*, 2011 ▸), it is not clear if a similar quality can be reached for germanium, given its much smaller use in the semiconductor industry and in synchrotron-based X-ray optics applications.

To gain more insight into this issue we carried out high-resolution rocking-curve imaging (RCI) on both of our germanium pieces to extract variations in *d*-spacing and the intrinsic crystalline quality. Measurements were carried out using a 2 mm × 11 mm (H × V) beam of 14.4 keV photons with a ∼1.5 meV bandwidth and angular divergence of ∼0.8 µrad. More information on this setup can be found in Shevyrtalov *et al.* (2021 ▸). The Ge(555) Bragg reflection was measured using a 2D photon-counting Lambda detector (X-Spectrum) with a 55 µm^2^ pitch and 256 × 256 pixels. The experiment was conducted at room temperature with an incidence angle of 2.4° (θ_B_ = 41.2° and *b* = −1/24), which gives a calculated extinction depth of 2.5 µm. In order to cover a large area of the crystals, the detector was moved vertically during the experiment.

**Figure 5:**
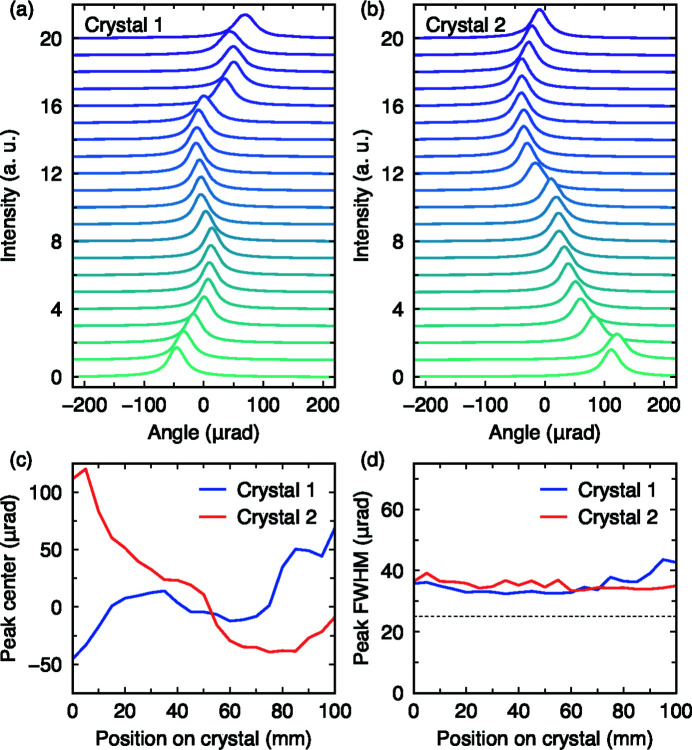
(*a*, *b*) The homogeniety of the Ge(111) C- and D-crystals was tested by conducting rocking scans of the (555) reflection in 5 mm steps along a ∼100 mm region. For clarity the maximum of each curve was normalized to unity. (*c*) Peak center and (*d*) FWHM as a function of crystal position were extracted by fitting the curves in (*a*, *b*). The dashed horizontal line in (*d*) is the expected theoretical value.

Measurements were made in 5 mm steps along each crystal to cover a 100 mm region. A region-of-interest covering 4 mm of the surface was then integrated to generate the Ge(555) RCI curves plotted in Figs. 5 ▸(*a*) and 5(*b*). Here a sizable variation in the peak position is clear on a macroscopic scale, indicating a non-uniform crystalline quality. To quantify this observation we treated each curve with a Lorentzian profile and plot the peak position trends in Fig. 5 ▸(*c*). The first crystal shows a peak-to-valley ratio (P/V) of 100 µrad for the Bragg angle, while crystal 2 shows a P/V of 150 µrad. This behavior can stem from two possible effects: bending of the lattice planes and/or differences in *d*-spacing. The former is unlikely since no clamping was applied — the crystals were mounted strain free — leaving us with lattice gradient. Shifts in Bragg angle can be related to *d*-spacing variation using 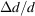 = 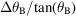 , giving ∼1–2 × 10^−4^ over an area of 100 mm. This value is at least three orders of magnitude worse than what is generally achievable with silicon. More promising trends are identified in the peak widths, which are plotted in Fig. 5 ▸(*d*). Crystal 1 shows a P/V of ∼5 µrad and a minimum FWHM of 33 µrad, which is only a factor of 1.3 larger than the theoretical value [dashed horizontal line in Fig. 5 ▸(*d*)]. In principle, this small discrepancy may stem from either the intrinsic Ge quality or a possible residual strain from polishing. In any case, these results show that the intrinsic resolving power of the spectrograph concept could be tested, as long as the X-ray footprint on the two Ge-crystals was minimized to avoid the macroscopic *d*-spacing deviations.

## 5. Spectrograph results   

**Figure 6:**
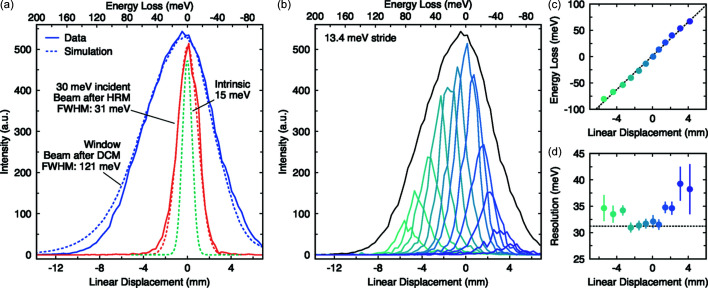
(*a*) Real world performance of the IRIXS Spectrograph (solid lines) is compared with ray-tracing simulations (dashed lines) for an incident beam *E*_0_ = 2840 eV and bandwiths of 600 meV and 30 meV. The intrinsic resolution was simulated by using an incident bandwidth of 1 meV. (*b*) The spectral response and mm-to-meV conversion was determined by systematically changing the incident energy in 13.4 meV strides and measuring the according shift in position on the detector. (*c*) The relationship between energy and position is linear, which was fitted, giving 16 meV mm^−1^. (*d*) The image remains in focus across the entire response function, although it degrades slightly at the edges of the window.

We can now present the experimental results of the IRIXS Spectrograph, which are shown in Fig. 6 ▸. Here, raw 2D images from the detector were binned along the dispersing direction and summed over the other axis. Following our RCI measurements the vertical size of the post-M_C_ beam was reduced from 5.5 mm to ∼0.1 mm using the slit system, resulting in a germanium footprint of <2 mm (the horizonal beam size of 5.5 mm was left intact). For each measurement in this configuration we exposed the detector for 50 min in order to have sufficient statistics for a comprehensive analysis of the spectral response. In Fig. 6 ▸(*a*) we start by comparing the spectrometer response using incident beams from the DCM (600 meV) and HRM (30 meV). The experimental data are compared with ray-tracing simulations run under the same conditions, which reveal an excellent agreement. The DCM beam results in a curve that covers 7 mm (FWHM) on the detector. In a clear demonstration of the spectrograph concept, the detected signal width narrows to 2 mm when the incident bandwidth is decreased to 30 meV with the HRM.

To experimentally determine the mm-to-meV conversion, the incidence HRM energy was scanned in steps of 13 meV, from around −80 meV to 60 meV, by rotating the sapphire HRM crystals. The data are shown in Fig. 6 ▸(*b*) along with the DCM curve. As expected the DCM signal forms an envelope over the HRM signal, representing the response function of the IRIXS Spectrograph. The HRM peak position is plotted as a function of incident energy in Fig. 6 ▸(*c*). The trend was fitted with a simple linear function from which a slope of 16 meV mm^−1^ was extracted, in good agreement with our 17 meV mm^−1^ estimate. With this number at hand we obtain a calculated FWHM of 121 meV and 31 meV for the DCM and HRM signals, respectively. We can compare the latter value with the simulated intrinsic spectrograph resolution of 15 meV [see Fig. 6 ▸(*a*)], which suggests that the instrument is primarily resolution limited by the incident bandwidth (*i.e.* the HRM) in the current design.

As pointed out by Sanchez del Rio & Shvyd’ko (2019 ▸), chromatic aberrations appear when mirrors (*i.e.* Montel optics) are used for focusing, as different energies have different focal points. Aberration-free images can, however, be collected if the detector is tilted to the focal plane. Here we identified the optimal angle by iterating our ray-tracing model. The peak width as a function of incident energy is plotted in Fig. 6 ▸(*d*), which confirms that this method works, revealing that the image remains sharp across the energy window of the spectrograph. Indeed, within 50 meV from the center the FWHM matches the theoretical value (horizontal dashed line), although the value starts to degrade slightly towards fringes of the spectrograph.

The results shown in Fig. 6 ▸ confirm that the IRIXS Spectrograph performs exceptionally well, with potential for future improvements (*e.g.* a better HRM and smaller slope error). It is worth noting that the simulations show an excellent agreement with the experimental results, despite not explicitly taking into account the intrinsic broadening of the germanium crystals as identified in Fig. 5 ▸(*d*). This could stem from the magnitude reduction in extinction depth with 2.840 keV photons, or simply because the Montel slope error contribution is slightly overestimated. The bigger challenge is to overcome the major macroscopic variations in *d*-spacing. To simulate the effect of our experimentally determined inhomogeneity, the germanium models were modified to introduce a variance 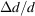 = 1 × 10^−4^. We found that the resulting divergence from the C-crystal increases from ∼6 to 60 µrad, effectively suppressing any collimation effect. This is then unfortunately amplified by the D-crystal by a factor of |*b*_3_| = −18 to a drastic value of 1000 µrad. Experimentally, we found that the HRM signal indeed broadened to cover the entire spectrograph window when the complete 5.5 mm beam was used. The most straightforward solution to this issue is to swap out the germanium crystals for high-quality silicon with a proven 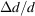 ≃ 1 × 10^−8^, and equivalent asymmetry parameters. As we mentioned previously, the reduced Darwin width in silicon means that a Si(111) D-crystal (*b* = −1/18) would not accept 120 meV but instead 60 meV. Such a change in material would therefore cut the energy window of the spectrum that can be imaged in a single-shot by half. The resolving power will, however, remain intact.

We finish by comparing the estimated spectral reflectivity of the spectrograph with the previous Rowland-type spectrometer which is based on a diced quartz ( 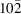 ) analyser (Gretarsson *et al.*, 2020 ▸). Using simulations we find that the reflectivity of the spectrograph is slightly better than in our old spectrometer, with a value of 1.3% versus 1.0%. It is quite remarkable that the reflectivity of the old spectrometer is not higher given that it has only one optical element. This can be understood since <5% of the area of a quartz pixel will Bragg reflect a monochromatic light (set by the Darwin width), leaving most of the pixel area unused. However, since the solid angle of the spectrograph is a factor of four smaller, the count rates would decrease by a factor of three. This is not insignificant but an acceptable trade-off for the improved energy resolution. Our prior experiments using the Rowland-type spectrometer showed that spectra of collective excitations at the Ru *L*_3_-edge can be collected in less than 10 min (Bertinshaw *et al.*, 2021 ▸). This means that, even if we consider the factor of six in the photon flux between nested and in-line HRM, meaningful high-resolution studies will be possible within a few hours. More generally, we envision that the RIXS spectrum can be collected either through a ‘snap-shot’ (for a limited energy range) or by tuning the incident energy (100–500 meV) — the small range for the latter option is not expected to change the resonance conditions.

## 6. Conclusion   

We have successfully designed and tested a first-of-its-kind ultra-high-resolution resonant inelastic X-ray scattering (RIXS) spectrometer at beamline P01 at the PETRA III Synchrotron, DESY. This spectrometer is based on the spectrograph concept first introduced for X-rays in Shvyd’ko (2015 ▸) and designed to operate at the Ru *L*_3_-edge (2840 eV). It uses a dispersive element combined with a focusing mirror to form an analyzer that images 120 meV of the RIXS spectrum with an intrinsic energy resolution of around 15 meV FWHM. In combination with a dispersionless nested four-bounce high-resolution monochromator we achieve an overall energy resolution better than 35 meV FWHM, in excellent agreement with ray-tracing simulations. Our work demonstrates that this novel approach to ultra-high-resolution tender RIXS is feasible, bridging the gap between ultra-high-resolution soft and hard X-ray RIXS, and providing an effective route to investigate the wide array of complex dynamics exhibited by Ru-based compounds.

## Figures and Tables

**Table 1:** 2840 eV Montel optic specifications

Mirror	AXO GmbH	Incoatec GmbH
Optical length (*L*)	150 mm	148 mm
Focal length (*S*)	200 mm	200 mm
Vertex radius (*p*)	0.53 mm	0.54 mm
Bragg angle (θ_B_)	2.1°	2.1°
Beam size (*b*)	5.5 mm × 5.5 mm	5.5 mm × 5.5 mm
Entrance aperture	3.8 mm	3.8 mm
Exit aperture	5.5 mm	5.5 mm
Multilayer material	Cr/C	Ni/C
Period (*d*_0_–*d*_1_)	4.98–7.85 nm	4.83–7.42 nm
Pair repetitions	80	100
Gamma ratio	0.35	0.45
Interlayer roughness (RMS)	≤0.3 nm	≤0.3 nm
Angular acceptance	30 mrad × 30 mrad	30 mrad × 30 mrad
Estimated divergence	∼100 µrad	∼100 µrad
Slope error (RMS)	≤12 µrad	≤12 µrad
Micro-roughness (RMS)	≤0.3 nm	≤0.3 nm
Estimated reflectivity	∼70%	∼70%
